# Asymptomatic Bacteriuria *Escherichia coli* Are Live Biotherapeutics for UTI

**DOI:** 10.1371/journal.pone.0109321

**Published:** 2014-11-18

**Authors:** Charles N. Rudick, Aisha K. Taylor, Ryan E. Yaggie, Anthony J. Schaeffer, David J. Klumpp

**Affiliations:** 1 Department of Urology, Feinberg School of Medicine, Northwestern University, Chicago, Illinois, United States of America; 2 Microbiology-Immunology, Feinberg School of Medicine, Northwestern University, Chicago, Illinois, United States of America; Cedars Sinai Medical Center, United States of America

## Abstract

Urinary tract infections (UTI) account for approximately 8 million clinic visits annually with symptoms that include acute pelvic pain, dysuria, and irritative voiding. Empiric UTI management with antimicrobials is complicated by increasing antimicrobial resistance among uropathogens, but live biotherapeutics products (LBPs), such as asymptomatic bacteriuria (ASB) strains of *E. coli*, offer the potential to circumvent antimicrobial resistance. Here we evaluated ASB *E. coli* as LBPs, relative to ciprofloxacin, for efficacy against infection and visceral pain in a murine UTI model. Visceral pain was quantified as tactile allodynia of the pelvic region in response to mechanical stimulation with von Frey filaments. Whereas ciprofloxacin promoted clearance of uropathogenic *E. coli* (UPEC), it did not reduce pelvic tactile allodynia, a measure of visceral pain. In contrast, ASB *E. coli* administered intravesically or intravaginally provided comparable reduction of allodynia similar to intravesical lidocaine. Moreover, ASB *E. coli* were similarly effective against UTI allodynia induced by *Proteus mirabilis, Enterococccus faecalis* and *Klebsiella pneumoniae*. Therefore, ASB *E. coli* have anti-infective activity comparable to the current standard of care yet also provide superior analgesia. These studies suggest that ASB *E. coli* represent novel LBPs for UTI symptoms.

## Introduction

Urinary tract infection (UTI) is the second-most common infectious disease, resulting in millions of cases in the U.S. annually and costing billions of dollars for associated treatment [1], [2]. The standard of care for UTI is antimicrobial therapy, where such therapy effectively sterilizes the urine of susceptible bacteria [3]. Unfortunately, uropathogenic bacteria are increasingly resistant to frontline antimicrobials, and multi-drug resistant strains are emerging rapidly [4], [5]. In addition to waning efficacy due to resistance, it is unclear that acute antimicrobial therapy is sufficient to prevent recurrent UTI. Data from murine models and clinical studies demonstrate that UPEC and other uropathogens establish quiescent reservoirs within bladder urothelium that can trigger subsequent acute infection [6]. These quiescent reservoirs are resistant to antimicrobial therapy, indicating a need for alternative approaches. While antimicrobial therapies are effective at sterilizing urine of susceptible bacteria, it is unclear that antimicrobial activity is the basis for symptomatic relief of UTI [4]. Indeed, recent studies in murine UTI suggest that symptom kinetics are mediated by initial receptor-mediated responses to bacterial surface molecules, not bacterial loads [7].

Given the rapid rise in antimicrobial resistance and the potential for collateral damage caused by conventional therapies [8]–[11], alternative approaches are needed, and live biotherapeutic products (LBPs) such as vaccines and probiotics offer the potential to treat UTIs without concerns about antimicrobial resistance. Lactobacilli are normally a major component of the vaginal microflora of fertile women that act as a natural barrier against ascending infection by uropathogens [12]. Clinical trials have demonstrated that vaginal application of *L. crispatus* reduces UTI occurrence, presumably by restoring or augmenting the natural protective barrier against ascending infection [13]–[15]. Alternatively, asymptomatic bacteriuria (ASB) *E. coli* have been harnessed to prevent UTI. Since the American Urologic Association Guidelines are to not treat ASB, ASB *E. coli* have been instilled into patients to occupy this biological niche and thereby prevent infection by “bacterial interference” [16]. Clinical inoculation of the prototypic ASB *E. coli* strain 83972 was shown to significantly reduce UTI among spinal cord injury patients with chronic indwelling or intermittent catheterization while protecting patients with incomplete bladder emptying from recurrent UTI [17]. Similarly, clinical trials with HU2117, a P fimbriae-deficient mutant of 83972, demonstrated significantly increased time between symptomatic UTI in neurogenic bladder patients [18]. These studies with 83972 suggest that ASB *E. coli* strains possess biologic activities that thwart uropathogens and/or alter UTI symptoms. These clinical studies were not designed to test the bacterial interference hypothesis directly so mechanism of ASB *E. coli* impact UTI pathogenesis is unknown.

We recently characterized pelvic pain responses in a murine model of UTI. Using tactile allodynia as a behavioral correlate to pelvic pain, we quantified pelvic allodynia in response to UPEC strain NU14 or ASB strain 83972. NU14 induced pelvic allodynia that was not observed in response to 83972, yet similar inflammatory responses were induced by either bacterial infection or by lipopolysaccharide (LPS) purified from either strain [7], [19]. Although neither instillation of 83972 nor its purified LPS induced pelvic allodynia, we found that 83972 LPS significantly reduced allodynia associated with NU14 infection, suggesting that ASB *E. coli* possess analgesic activity [19]. Here, we evaluated ASB *E. coli* strains for efficacy treating colonization of the urinary tract by uropathogens and for efficacy treating pain associated with UTI. We found that ASB *E. coli* exhibit anti-infective and analgesic activities in murine UTI, suggesting a potential use of ASB *E. coli*-based LBPs for treating UTI and other symptom-based disorders.

## Methods

### Animals

Adult female mice C57BL/6J 10–14 weeks of age were purchased from Jackson Laboratory. All experiments were performed using protocols approved by Northwestern University Animal Care and Use Committee as previously described [7], [19].

### Bacterial strains

NU14 is a clinical isolate of *E. coli* originally obtained from the urine of a cystitis patient and is considered archetypal for UPEC [20]. ASB strain 83972 was isolated from a young Swedish girl who was infected for at least 3 years without symptoms [21], [22] and is one of the most extensively characterized ABS strains [23], [24]. ASB *E. coli* strains were previously collected as a panel of consecutive clinical isolates [25] or isolates from the Northwestern Urology Clinic for the purposes of this study. *Proteus mirabilis*, *Enterococcus faecalis* and *Klebsiella pneumoniae* were isolated from discarded urine of uncomplicated cystitis patients. All clinical isolates obtained for this study were identified by standard microbiologic testing, including Gram staining and Microscan (Siemens Healthcare). Bacteria were cultured by serial passage in Luria broth at 37° in static broth [26], [27]. *E. coli* strains were differentiated by plating onto selective agar: NU14 is resistant to streptomycin, 2–12 is resistant to streptomycin/ampicillin, and 83972 is resistant to chloramphenicol.

### Infection and behavioral testing

Female mice were anesthetized with isoflurane and instilled via transurethral catheter with a volume of 10 µl containing 1×10^8^ CFU bacteria in saline [28]. Following termination of the experiment the bladders were harvested, homogenized and plated on agar with or without antibiotic selection for colonization. Mice were tested prior to bacterial infection (baseline) and up to PID 20. Referred hyperalgesia and tactile allodynia was tested, as previously described [29], [30], using von Frey filaments applied to the abdomen [29], [30].

### Treatments

Lidocaine was administered as a 2% lidocaine solution in water that was instilled via a Hamilton syringe and polyethylene catheter as previously described [30]. Ciprofloxacin (10 mg/kg/0.15 ml) drug therapy was administered daily as a 3-day course via I.P. via injection.

### Statistical analyses

Colonization, inflammation and behavioral data were analyzed with the student t-test or a Kruskal-Wallis test followed by Dunn's post test or repeated ANOVA followed by a Dunnett's post test using Prism software from GraphPad Inc. as appropriate. A value of P<0.05 was considered statistically significant.

### Ethics statement

These studies were conducted under Northwestern University IACUC protocols 2010–1788 and 2009–1677.

## Results

### ASB isolate 83972 attenuates UTI allodynia and bacteriuria

**Figure 1:**
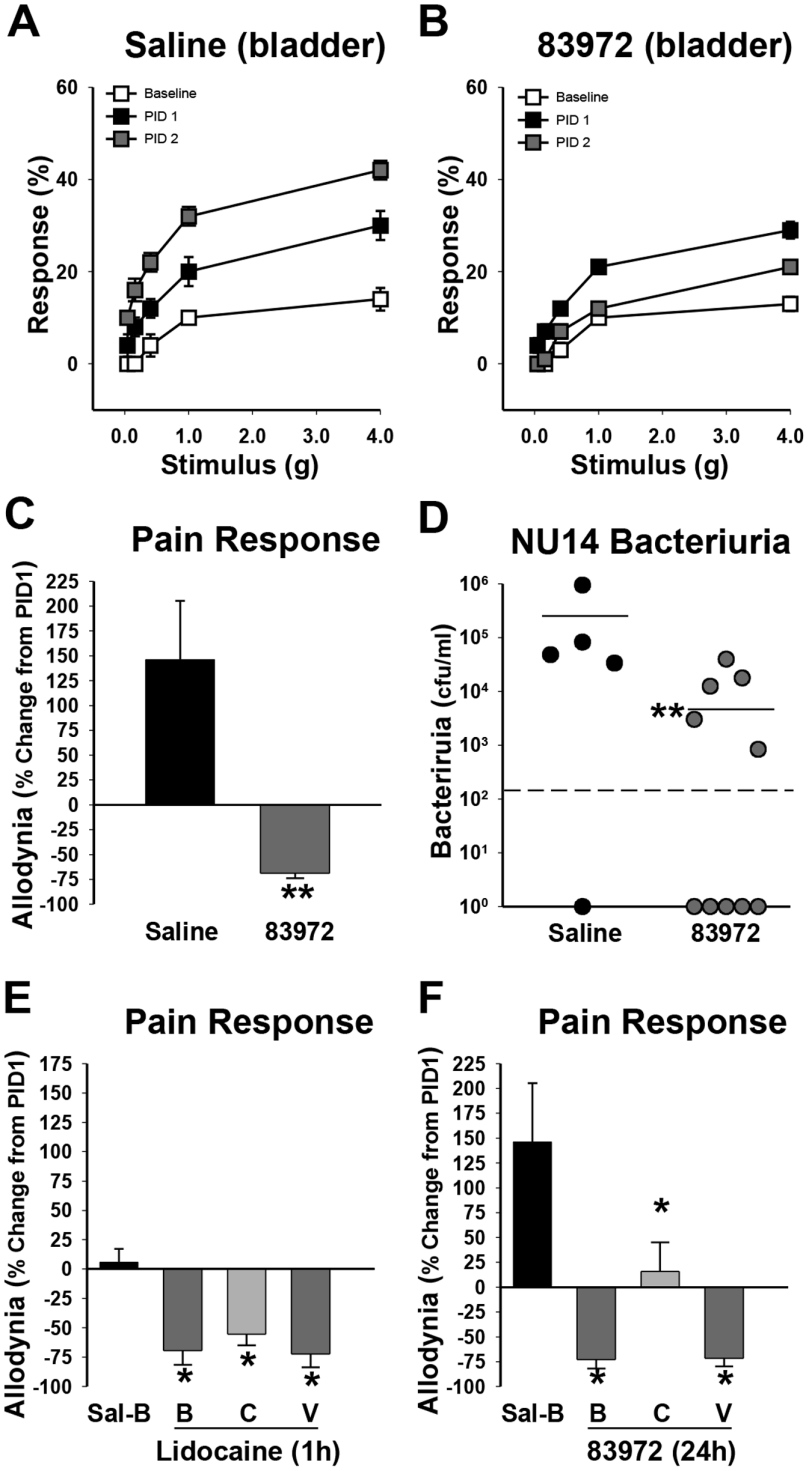
*E. coli* 83972 attenuates NU14-induced bacteriuria and pelvic pain. A) Referred visceral hyperalgesia was measured as responses to mechanical stimulation of the pelvic region with von Frey filaments of 5 intensities. Responses were quantified at baseline, PID1 following NU14 infection, and 24 hours following of saline (n = 5) or 83972 (n = 10) instillation (PID2). B) NU14-infected mice exhibited a significant decrease in allodynia 24 hours following 83972 instillation. C) Percent increase in allodynia from PID1 was 146.2% in saline-treated mice, but decreased by over 68.9% in 83972-treated mice (P<0.01). D) Mice treated with 83972 exhibited a significantly less NU14 bacteriuria compared to saline-treated mice (P<0.01). Dashed line represents limit of detection. E) At PID1, NU14-infected mice were instilled with saline into the bladder or 2% lidocaine in the bladder, vaginal introitus or colon. Allodynia was significantly reduced at 1 h after lidocaine into any compartment, relative to bladder saline (*P<0.05; n = 9 saline, n = 10 bladder lidocaine, n = 11 colon lidocaine, n = 12 vaginal lidocaine). F) NU14-infected mice exhibited increased allodynia 24 hours after bladder instillation of saline, relative to PID1 (n = 10). Allodynia was significantly decreased at 24 hours following 83972 instillation into the bladder, colon or vaginal introitus (*P<0.05, n = 10 all groups). Data are reported as the mean ± SEM (A–C, E & F).

Since 83972 LPS attenuated UTI-associated allodynia [19], we hypothesized that intact 83972 bacteria would exhibit similar analgesic activity. To test this, female B6 mice were instilled with the UPEC strain NU14 via transurethral catheter to initiate UTI; after 24 h, mice were instilled with saline or 83972. Allodynia was quantified at baseline and daily throughout the experiment by assessing responsiveness to stimulation of the pelvic region with a graded series of von Frey filaments [30]. Saline-treated mice exhibited significant pelvic allodynia (Figs. 1A and C) relative to 83972-treated mice that showed a significantly reduced allodynia (Figs. 1B and C, P<0.01). Since 83972 is thought to prevent UTI by bacterial interference, we also quantified bacteriuria. 83972 significantly reduced urinary NU14, relative to saline instillation (Fig. 1D, P<0.01).

### ASB isolate 83972 mediates analgesia via crosstalk

We have shown previously that colonic lidocaine attenuates pelvic pain in a neurogenic cystitis model as effectively as intravesical lidocaine, demonstrating organ crosstalk in pelvic pain relief [30]. We used a similar strategy to evaluate whether analgesic activity of ASB *E. coli* was capable of crosstalk during UTI. Allodynia was quantified 1 hour after lidocaine administration to mice that were infected with NU14 24 h prior (Fig. 1E); as a result, allodynia was not increased in the saline group (compare saline group in Fig. 1F). Relative to bladder instillation of saline, 2% lidocaine significantly and similarly attenuated UTI allodynia whether administered into the bladder, colon, or vagina (Fig. 1E, P<0.05). Analgesic activity of 83972 was evaluated at 24 h, and colonic 83972 significantly reduced saline-treated UTI allodynia (Fig. 1F, P<0.05), consistent with our previous findings for colonic modulation of cystitis pain [30]. 83972 significantly and similarly attenuated NU14-induced allodynia when administered intravesically or intravaginally (P<0.05). Remarkably, 83972 analgesic activity was comparable to lidocaine, whether acting directly or via crosstalk from the vagina (compare Figs. 1E and F).

### ASB isolate 83972 attenuates bacteriuria comparable to ciprofloxacin

**Figure 2:**
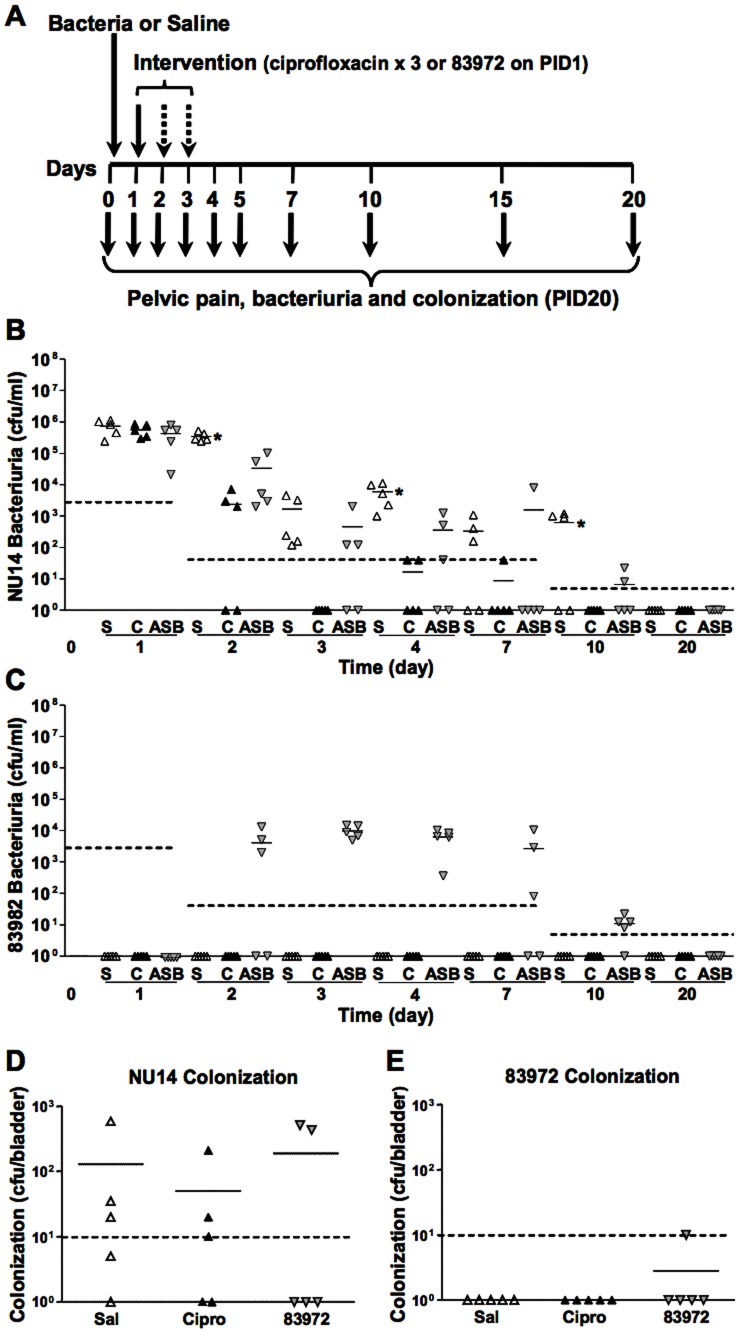
*E. coli* 83972 attenuates NU14 bacteriuria. A) Experimental scheme for assessing efficacy of a single administration of ASB therapy relative to 3-day course of ciprofloxacin (n = 5 for all groups). B) Mice infected with NU14 exhibited a significant decrease in NU14 bacteriuria 24 hours after initiation of ciprofloxacin (group C, back triangles, PID2, 4, 10), relative to saline-treated mice (group S, white triangles, *P<0.05). A single instillation of 83972 (group ASB, grey inverted triangles) also resulted in a significant decrease in NU14 bacteriuria after 24 hours (PID2, 4, 10, *P<0.05). C) Urinary 83972 for all three groups during the experiment. D and E) Bladder colonization on PID20 was not significantly different between the groups tested. Dashed lines represent limits of detection.

Since 83972 significantly reduced NU14 bacteriuria (Fig. 1D), we evaluated the anti-infective activity of intravesical 83972 relative to a three-day course of ciprofloxacin, a standard clinical regimen for uncomplicated UTI. All three groups exhibited similar levels of bacteriuria at post-infection day 1 (PID1) and had sterile urine by PID20 (Fig. 2B). Mice receiving ciprofloxacin or 83972 exhibited significantly reduced NU14 bacteriuria from PID2, 4 and 10, compared to the saline group (Fig. 2B, P<0.05). 83972 also became transiently established in the urinary tract, as indicated by 83972 bacteriuria from PID2 to PID10 (Fig. 2C). At PID20, most mice exhibited low levels of residual bladder colonization consistent with previous reports [31], no differences in NU14 colonization were observed between bladders of treated and untreated mice, and 83972 was largely cleared (Figs. 2D and E). While not significantly different here, we cannot exclude the possibility that ciprofloxacin therapy would exhibit increased antimicrobial activity relative to 83972 with larger samples. Nonetheless, these data suggest that the antimicrobial activity of ciprofloxacin and the anti-infective activity of 83972 are comparable and are manifested at the level of bacteriuria without fully sterilizing the bladder of bacterial reservoirs.

### ASB *E. coli* differentially attenuate UTI allodynia

**Figure 3:**
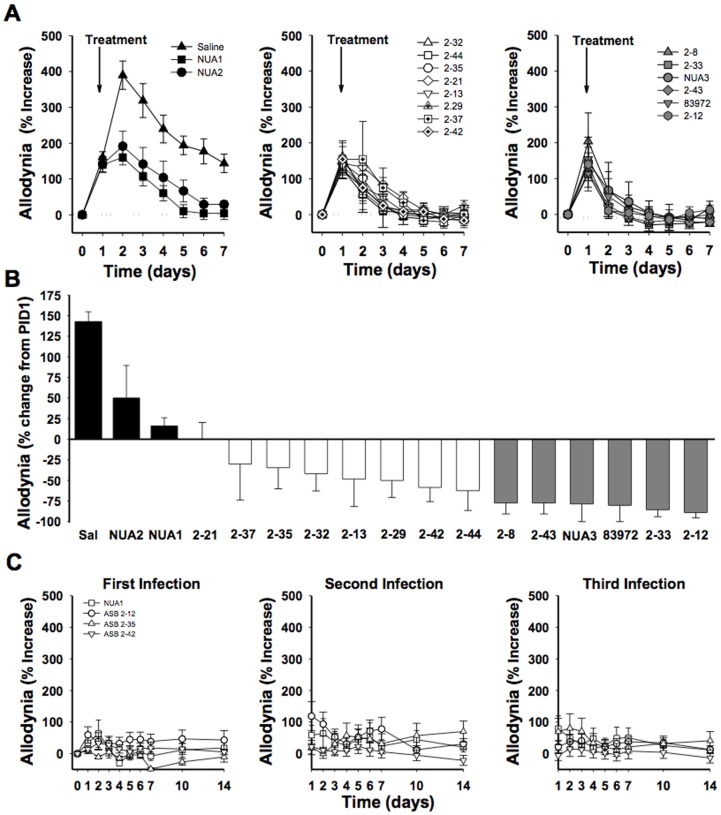
ASB strains differentially attenuate UTI visceral pain. Modulation of UTI visceral pain by a panel of 16 ASB *E. coli* strains. A) Mice were infected with NU14 and then instilled with saline or ASB *E. coli* at PID1, and allodynia was quantified through PID7 (n = 6 for all groups). For clarity, timecourses of allodynia for all 17 conditions were divided among 3 panels. All 16 ASB strains attenuated NU14-induced pelvic pain compared to the saline treated group. B) The relative analgesic activity of ASB strains was arbitrary grouped according to the magnitude of analgesia: strains with analgesic relative to saline but increased allodynia from PID1 (black bars), those that exhibited no increase in allodynia from PID1 to less than 75% reduction in visceral pain from PID1 (white bars), and those that exhibited greater than 75% reduction (gray bars). C) Representative ASB strains from each group in (B) were used in serial infections at two-week intervals. Serial infection did not induce allodynia. Data are reported as the mean ± SEM.

To determine whether analgesic activity was a unique property of 83972, we screened a panel of ASB *E. coli* strains including 3 strains collected from the Northwestern Urology Clinic for this study and 12 ASB strains from a panel of 54 consecutive clinical isolates [25]. As in Figure 1, mice were instilled with NU14 and then instilled with saline or an ASB panel strain 24 hours later and evaluated for pelvic responsiveness prior to instillation, 24 hours after NU14 instillation, and daily after saline or ASB treatment; strains were then grouped arbitrarily by relative analgesic activity (panels of Fig. 3A). All ASB isolates attenuated NU14-induced pelvic allodynia when compared to saline-instilled controls, but the magnitude of attenuation varied (Figs. 3A & B). While some mice exhibited an increase in pelvic pain 24 hours after infection with an ASB isolate (Fig. 3A, left panel), indicating only modest analgesic activity, they were significantly decreased compared to the saline-instilled control (Fig. 3B, black bars, P<0.05). Eight strains exhibited an intermediate analgesic phenotype, ranging from no increase in allodynia from PID1 to 70% reduction in allodynia (Fig. 3A, middle panel, and Fig. 3B, white bars). A third group was comprised of 6 ASB isolates that displayed a high analgesic phenotype, relative to PID1, attenuating cystitis allodynia from 75% to 85% (Fig. 3A, right panel, and Fig. 3B, grey bars). Since we recently showed that serial infections with could reveal a pain phenotype in an *E. coli* strain otherwise exhibiting no pain phenotype [7], three serial infections were performed with strains from the ASB panel (Fig. 3C). None of the ASB isolates tested induced pain following serial infections, consistent with our recent report that serial infection with 83972 did not induce pain [7]. These data demonstrate that ASB *E. coli* exhibit a range of analgesic phenotypes, and that the prototypic ASB strain, 83972, possesses a highly analgesic phenotype. However, since strain 2–12 had the greatest analgesic activity against cystitis pain, 85%, we focused on 2–12 for further study.

### ASB isolate 2–12 is superior to ciprofloxacin for analgesia

**Figure 4:**
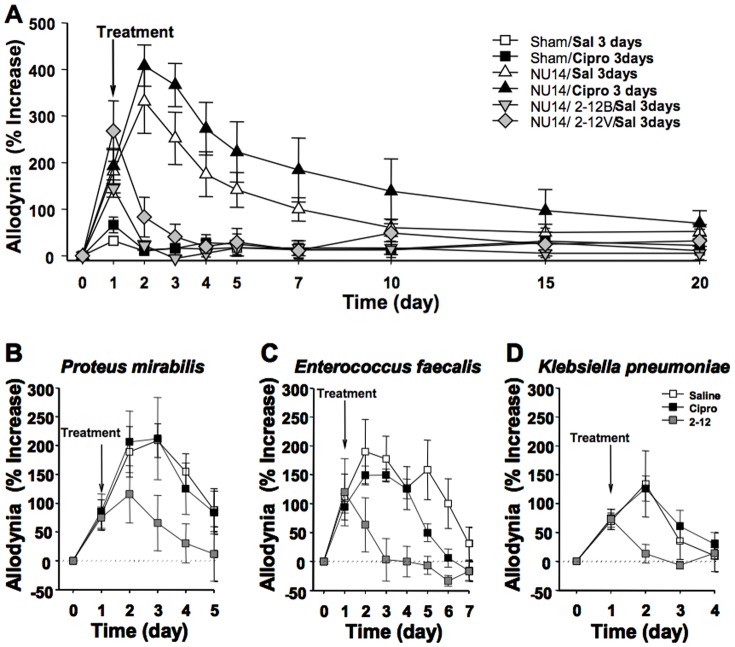
ASB *E. coli* isolate 2–12 rapidly attenuates UTI visceral pain of diverse uropathogens. A) Allodynia was quantified daily in groups of sham– or NU14-infected mice that were then treated at PID1 with saline, a three-day course of ciprofloxacin, or a single dose of intravesical or intravaginal 2–12 as described in Figure 2A (n = 8 in all groups except the 2–12 intravaginal group where n = 6). NU14-infected mice exhibited a significant decrease in pelvic pain 24 hours following treatment with 2–12 via the bladder or vaginal introitus (P<0.05). Allodynia was quantified in mice infected via transureathral catheter with *Proteus mirabilis* (PM1), *Enterocccus faecalis* (EF1) and *Klebsiella pneumoniae* (KP1) and then treated with a three-day course of ciprofloxacin initiated at PID1 or a single intravesical dose of 2–12 (n = 5). B) PM1 induced pelvic allodynia that was significantly attenuated on PID3 and PID4 by 2–12 treatment, relative to the ciprofloxacin or saline groups (P<0.05). C) EF1 induced allodynia that was significantly attenuated on PID2-PID4 by 2–12 treatment, relative to ciprofloxacin or saline groups (P<0.05). Allodynia was also significantly reduced on PID5–6 compared to the saline group (P<0.05). D) KP1 induced allodynia that was significantly attenuated on PID2 by 2–12 treatment, relative to ciprofloxacin or saline groups (P<0.05).

We evaluated the effect of 2–12 on NU14-induced cystitis pain, relative to sham treatment or a 3-day course of ciprofloxacin using the schemed depicted in Figure 2A. Since we observed that 83972 could exert analgesic activity via crosstalk (Fig. 1F), we also evaluated 2–12 administered intravesically and intravaginally. Control groups instilled with saline then treated with 3 injections of saline or ciprofloxacin did not develop detectable allodynia (Fig. 4A). Consistent with previous observations [19], mice instilled with NU14 developed allodynia that decayed over two or three weeks, but ciprofloxacin therapy did not reduce allodynia better than saline during the course of the experiment (Fig. 4A). In contrast, mice receiving a single therapeutic dose of ASB isolate 2–12 exhibited a rapid and significant decrease in allodynia, whether that dose was administered to the bladder or vaginal introitus (Fig. 4A, P<0.05).

### ASB isolate 2–12 is a superior analgesic against diverse uropathogens

Since ASB *E. coli* strains possess anti-infective and analgesic activity against UPEC, we examined effects against non-*E. coli* uropathogens. Allodynia was quantified in murine models of UTI Gram-positive bacteria, and responses to 2–12 therapy were compared with saline and ciprofloxacin. A single clinical isolate of *Proteus mirabilis* (PM1, Gram negative), *Enterocccus faecalis* (EF1, Gram positive) and *Klebsiella pneumoniae* (KP1, Gram negative) was obtained from urines of the Northwestern Urology Clinic and typed using standard microbiologic procedures. PM1-induced acute pelvic allodynia that was unaltered by a 3-day course of ciprofloxacin (Fig. 4B). However, PM1-induced allodynia was significantly attenuated in mice instilled with 2–12 on PID3 and PID4 (P<0.05). Similarly, 2–12 significantly reduced allodynia associated with EF1 or KP1 infection (Figs. 4C and D, respectively, P<0.05). Thus, 2–12 exhibits superior analgesic activity to current antimicrobial therapy in multiple urine UTI models.

## Discussion

Our findings demonstrate that ASB *E. coli* 83972 exhibit anti-infective activities comparable to ciprofloxacin in a murine UTI model. While ciprofloxacin promoted clearance of uropathogenic *E. coli* (UPEC), it did not reduce pelvic tactile allodynia, a measure of visceral pain. In contrast, ASB *E. coli* administered intravesically or intravaginally provided comparable reduction of allodynia similar to intravesical lidocaine. Moreover, ASB *E. coli* were similarly effective against UTI allodynia induced by *Proteus mirabilis, Enterococccus faecalis* and *Klebsiella pneumoniae*. Therefore, ASB *E. coli* have anti-infective activity comparable to the current standard of care yet also provide superior analgesia suggesting that ASB *E. coli* represent novel LBPs for UTI..

UTI is associated with a leukocytic influx dominated by neutrophils to drive clearance of UPEC [32], [33]. Quantifying urinary myeloperoxidase (MPO) as a marker of bladder neutrophil influx, we previously found that 83972 and UPEC strain NU14 similarly induce MPO and inflammation [7], [19]. We also observed that purified LPS preparations from 83972 and NU14 resulted in similar levels of neutrophil recruitment when instilled into the bladder and similarly activated splenic macrophages in culture [19], so it is unlikely that 83972 markedly enhances neutrophil influx. Another potential mechanism is the rapid urothelial apoptotic host defense response that is induced by the interaction of the UPEC type 1 pilus adhesin (FimH) with uroplakins [20], [34]–[36], however 83972 do not express type 1 pili. We speculate that one anti-infective effect of ASB *E. coli* is to function as an adjuvant that enhances immune responses to uropathogens.

Ciprofloxacin and other antimicrobials sterilize the urine of susceptible bacteria, but the relationship between antimicrobial activity and symptomatic relief is increasingly unclear. A recent study revealed that ibuprofen was as effective as ciprofloxacin at ameliorating symptoms of dysuria and frequency during acute, uncomplicated UTI [37]. This is consistent with previous observations that UTI symptoms resolve spontaneously in the majority of patients and do not correlate with urinary bacterial loads [38], [39]. We observed that ciprofloxacin did not alter pelvic allodynia in murine UTI (Fig. 4A), reinforcing clinical findings and consistent with our recent report that pelvic allodynia can persist long after bacterial clearance [7]. In contrast, we find ASB *E. coli* significantly reduce UPEC-induced allodynia, suggesting analgesic activity that is not unique to a single ASB strain but rather but varies across a spectrum. We find several aspects of this analgesic effect remarkable. First, ASB *E. coli* can exhibit analgesic activity comparable to intravesical lidocaine (Figs. 1E and F), suggesting clinical utility. Second, the significant reduction in allodynia when 83972 was administered in the vagina or colon indicates that the analgesic activity against UPEC can be mediated via organ crosstalk, similar to lidocaine (Fig. 1E and F; [30]). The physiologic basis for organ crosstalk is visceral convergence of sensory pathways among pelvic organs such as the bladder and bowel [40]–[42] and this observation of organ crosstalk in ASB *E. coli* analgesia is evidence that ASB *E. coli* may mediate effects independent of bacterial interference. Since prior clinical studies of 83972 and HU2117 demonstrated significant reduction in symptomatic events in patients prone to recurrent UTI [17], [18], [43], an alternative explanation for those findings is that 83972 analgesic activity masked symptoms that would have been scored as a symptomatic recurrence. Third, 2–12 reduced allodynia induced by infection with non-UPEC uropathogens, demonstrating that ASB *E. coli* exhibit analgesic activity against diverse insults. Since we reported that LPS purified from 83972 reduced allodynia in NU14 infection [19], it is likely that the analgesic activity of other ASB *E. coli* strains is generally mediated by LPS through the cognate receptor TLR4 [7], [19].

The anti-infective and analgesic properties of ASB *E. coli* described here suggest several clinical applications, in addition to the use as a prophylactic for catheterized patients as reported previously [16], [43]. The anti-infective activity of 83972 was comparable to ciprofloxacin in promoting clearance for acute UTI, suggesting that ASB *E. coli* could be administered as a therapy of uncomplicated UTI. In both acute and recurrent UTI, the analgesic effect would be an advantage of ASB *E. coli* therapy over conventional antimicrobials. Finally, we observe that ASB *E. coli* reduced allodynia associated with diverse acute insults, suggesting general analgesic effects could be exploited to treat non-infectious pain conditions. Patients suffering chronic pelvic pain of interstitial cystitis/bladder pain syndrome (IC) can gain temporary relief from intravesical lidocaine [44]. Since ASB strains exert analgesic activity comparable to lidocaine and mediate these effects via organ crosstalk, ASB *E. coli* therapy could be used for chronic pelvic pain conditions such as IC, vulvodynia, or endometriosis.

The U.S. FDA classifies whole, live microorganisms that exhibit drug activities as LBPs. LBPs are subject to FDA oversight using the same Investigational New Drug regulatory pathway as small molecule drugs [45]. Given that ASB *E. coli* are considered harmless and clinical guidelines are to not treat ASB, therapeutic use of ASB *E. coli* would be safe. We find that ASB *E. coli* is cleared from the urinary tracts of healthy mice within days and consistent with clinical studies revealing the prevalence of ASB among healthy young women is approximately 5% [46]. Thus, the urinary tract appears similarly resistant to stable colonization by ASB *E. coli* in both healthy young women and mice, suggesting that ASB *E. coli* would be safe for clinical use as an LBP. ASB *E. coli* therapy also would not contribute to resistance against conventional antimicrobials. Finally, ASB *E. coli* address unmet needs for effective therapies against recurrent UTI and chronic pelvic pain, representing novel LBP candidates for urologic disease.
